# Convenient and efficient *N*-methylation of secondary amines under solvent-free ball milling conditions

**DOI:** 10.1038/s41598-024-59374-z

**Published:** 2024-04-16

**Authors:** Mikołaj Walter, Olga Ciupak, Karol Biernacki, Janusz Rachoń, Dariusz Witt, Sebastian Demkowicz

**Affiliations:** Department of Organic Chemistry, Faculty of Chemistry, Gdańsk University of Technology, Narutowicza 11/12, 80-233 Gdansk, Poland

**Keywords:** Mechanochemistry, Vibrational ball mill, Amines, *N*-methylation, Solvent-free synthesis, Organic chemistry, Synthetic chemistry methodology, Reaction mechanisms, Synthetic chemistry methodology, Solid-phase synthesis

## Abstract

In the present work, we report the development of a rapid, efficient, and solvent-free procedure for the *N*-methylation of secondary amines under mechanochemical conditions. After optimization of the milling parameters, a vibrational ball mill was used to synthesize 26 tertiary *N*-methylated amine derivatives in a short time of 20 min (30 Hz frequency) and high yields ranging from 78 to 95%. An exception was compounds having a hydroxyl group in their structure, for which a decrease in reaction efficiency was observed. During our research, we investigated alternate reaction selectivity occurring in compounds able to form ring closure products that are 3,4-dihydro-2*H*-1,3-benzoxazine derivatives instead of *N*-methylated products. The liquid-assisted grinding technique has been applied using formalin as a methylating agent and sodium triacetoxyborohydride as a reducing agent in a reductive amination reaction.

## Introduction

In recent years, many attempts have been made to eliminate or at least strictly limit the necessity for the usage of solvents in the chemical industry. Solutions for such problems range from the exchange of organic solvent for water or carrying out the reactions in the solid state. The three most important techniques, applied to minimize environmental impact and reduce energy consumption, are microwave-assisted synthesis, ultrasound-assisted synthesis, and mechanochemistry^1^. Of those three, mechanochemistry is probably the least known yet apparently the most promising. According to IUPAC, the reaction is considered mechanochemical when the energy applied to the reagents is generated in a mechanical process^2^, such as grinding in a ball mill. The involvement of a mechanical process usually makes it possible to conduct a reaction without the presence of any liquid, which is highly problematic in conventional organic synthesis. Most laboratory-scale experiments are performed with a mixer or planetary ball mills. Conducting chemical processes using mechanochemical methods has a limited impact on the natural environment and is classified as a “green chemistry approach”. Furthermore, ball milling synthesis is also associated with significant advantages over traditional heating as well as over other green methods. Due to the high concentration of molecules, ball milling techniques often result in a shortening of the reaction time^3–5^ but also in a difference in selectivity^6^. Conducting reactions by mechanical grinding can also lead to different products and allow the synthesis of compounds that do not seem to be generated under conventional solvent-based methods^7–9^. Moreover, ball-milling organic synthesis is characterized by a much lower energy demand than any other synthetic technique. The separation of desired products from reaction mixtures is often facilitated due to the possibility of reacting stoichiometric amounts of substrates followed by simple washing of the product with water. Mechanochemistry has already been applied in some areas of the pharmaceutical industry, but it is mostly applied to solid form screening, such as the formation of cocrystals, salts, and polymorphs^10–12^. On the other hand, the number of active pharmaceutical ingredients (APIs) synthesized mechanochemically is underwhelming. Recently, a multistep mechanochemical protocol has been used to synthesize compound PZ-1190, a multitarget ligand for serotonin and dopamine receptors with potential antipsychotic activity in rodents^13^. The mechanochemical reductive alkylation was involved in the developed protocol which improved the overall yield, reduced the reaction time, and decreased the use of toxic reagents and organic solvents. Another feature of mechanochemistry that has been advantageous over the conventional synthesis of APIs is the possibility of avoiding the usage of external catalysts and instead replacing those catalysts with milling vessel and ball materials^14^. The advantages of mechanochemical synthesis together with the lack of effective solvent-free synthetic procedures, make it desirable or even necessary to intensify the research on ball-milling chemistry.

The largest influence on mechanochemical API synthesis was the development of the Liquid Assisted Grinding (LAG) technique^9,15,16^. LAG involves the ball milling of solid-state reagents with the addition of a small amount of liquid^17^. The reaction is considered mechanochemical if the amount of liquid does not exceed 2 μL per milligram of reagents. In conventional solution, the amount of liquid typically reaches over 12 μL per milligram. The presence of liquid in mechanochemical reactions enhances the reactivity and can also affect the selectivity of the reaction. Amines are one of the most abundant classes of organic compounds. The functionalization of amines, such as methylation, is an important step in the synthesis of pharmaceuticals, dyes, or detergents. Although it may seem like introducing a small methyl group into a large organic substance does not have a notable impact on the properties of the molecule, there is substantial evidence that parameters such as lipophilicity can be drastically altered^18,19^. Over the last few decades, various diverse methods have been developed for efficient *N*-methylation of secondary amines and for mono- or dimethylation of primary amines. The main variation in these procedures is the nature of the methylating agent. The most frequently used agents are methyl iodide and dimethyl sulfate, both of which are extremely carcinogenic and environmentally hazardous. Another well-known example of the *N*-methylation method is the Eschweiler-Clarke reaction employing formaldehyde and formic acid, and its modified version known as reductive amination, where sodium borohydrides are used instead of formic acid. Several green chemistry strategies, including the use of methanol or dimethyl carbonate, have also been found to effectively methylate amine derivatives^20–23^. Nevertheless, to date, no procedure that represents a solvent-free *N*-methylation reaction under mechanochemical conditions has been described.

The present study was performed to develop an efficient, solvent-free procedure for mechanochemical *N*-methylation of secondary amines under reductive amination conditions. After optimization of the process, we synthesized 26 derivatives with organic solvent used only for the extraction process. Additionally, the effect of applying mechanical energy to the reactions was investigated in terms of selectivity toward generated products.

## Materials and methods

### General methods and materials

Secondary amines were synthesized according to the described procedure^24^. The appropriate aldehyde and primary amine derivatives were commercially available from Sigma Aldrich. Formaldehyde was available as a 37% solution in water from Fisher Bioreagents. Sodium triacetoxyborohydride was purchased from Apollo Scientific. Reactions were accomplished in an Anton Paar BM500 vibrational ball mill by using stainless-steel milling beakers (5 mL) and stainless-steel milling balls. ^1^H NMR spectra were recorded on a Varian Unity Inova 500 (500 MHz) spectrometer. Chemical shifts δ are reported in parts per million relative to the residual solvent peak (DMSO-d_6_ = 2.49 ppm for 1 H). Coupling constants are given in Hertz. High resolution mass spectra were recorded on Agilent 6545 Q-TOF. Thin-layer chromatography (TLC) was carried out with Polygram SIL G/UV254, silica gel (Macherey–Nagel GmbH & Co. KG, Duren, Germany). Compounds were visualized by means of irradiation with UV light and/or by treatment with a solution of ninhydrin. Flash chromatography was carried out using Büchi FlashPure Cartridges (silica gel 40 μm irregular) on a Büchi Pure Chromatography System with an integrated UV and ELSD detector. Melting points (uncorrected) were determined using a Stuart Scientific SMP30 apparatus. Infrared (IR) spectra were recorded using a Nicolet 8700 spectrometer.

### General method for the mechanochemical synthesis of *N*-benzyl-*N*-methylaniline derivatives

A ball-mill vessel was charged with *N*-benzylaniline hydrochloride (110 mg, 0.5 mmol, 1 equiv), sodium carbonate (53 mg, 0.5 mmol, 1 equiv), formalin (75 μL, 1 mmol, 2 equiv) and sodium triacetoxyborohydride (318 mg, 1.5 mmol, 3 equiv). One stainless-steel ball with a 10 mm diameter was added, and milling was performed at 30 Hz for 20 min. The reaction mixture was then extracted with dichloromethane (5 mL). After the evaporation of the solvent, the crude product was purified by flash chromatography on silica gel (hexane/DCM 100:0 → 80:20). The identity of the product was confirmed by spectroscopic analysis.

### General method for the solvent-based synthesis of *N*-benzyl-*N*-methylaniline derivatives

A mixture of *N*-benzylaniline hydrochloride (105 mg, 0.5 mmol, 1 equiv), sodium carbonate (53 mg, 0.5 mmol, 1 equiv), formalin (75 μL, 1 mmol, 2 equiv) and sodium triacetoxyborohydride (318 mg, 1.5 mmol, 3 equiv) was added to the flask containing acetic acid (5 ml). The resulting mixture was stirred at ambient temperature over 20 min, followed by evaporation of acetic acid under reduced pressure. The reaction mixture was then extracted with dichloromethane (2 × 15 mL). After the evaporation of the solvent, the crude product was purified by flash chromatography on silica gel (hexane/DCM 100:0 → 80:20). The identity of the product was confirmed by ^1^H NMR spectroscopic analysis.

## Results and discussion

### Optimization of the process

First, we decided to test the impact of the number and diameter of the milling balls on the efficiency of the *N*-methylation reactions. The total mass of the milling balls was always approximately equal to the mass of one ball with a 10 mm diameter. In our study, we observed that increasing the diameter of the balls, thereby decreasing their number, correlated with an increase in reaction yield. The next parameter we tested was the milling time, and we found that carrying out the reaction for 20 min was optimal since longer milling did not provide higher efficiency. Following these results, we decided to evaluate the effects of various milling frequencies on the progress of the reaction and found that the best results were obtained when applying a 30 Hz frequency. After all milling parameters were determined, we decided to test various borohydrides, including sodium borohydride, sodium cyanoborohydride, and sodium triacetoxyborohydride, which acted as reducing agents. In this study, we found that the use of sodium triacetoxyborohydride resulted in the best outcomes. Reduction reactions with sodium borohydride and sodium cyanoborohydride resulted in a mixture of undefined products and starting material, presumably because of the reduction of formaldehyde under mechanochemical conditions. The last aspect we investigated was the form of formaldehyde that was applied to the reaction. Unfortunately, solid-state paraformaldehyde did not seem to react under the given conditions, so all of the reactions were carried out with the use of formalin. Additionally, after optimizing all milling conditions, we carried out the reaction with a mortar and pestle, which resulted in an unsatisfactory yield of the product or no reaction. The optimization results are collected in Table 1.

**Table 1:** Optimization of the milling parameters.


Number of balls (–)	Ball diameter (mm)	Time (min)	Frequency (Hz)	Reducing agent (–)	Aldehyde form (–)	Mass of the product (mg)	Yield (%)
1	10	15	30	NaBH(OAc)_3_	Formalin	85	80
3	7	15	30	NaBH(OAc)_3_	Formalin	67	63
8	5	15	30	NaBH(OAc)_3_	Formalin	43	40
31	3,2	15	30	NaBH(OAc)_3_	Formalin	38	36
1	10	10	30	NaBH(OAc)_3_	Formalin	61	57
1	10	20	30	NaBH(OAc)_3_	Formalin	90	84
1	10	25	30	NaBH(OAc)_3_	Formalin	90	84
1	10	20	25	NaBH(OAc)_3_	Formalin	71	67
1	10	20	20	NaBH(OAc)_3_	Formalin	61	57
1	10	20	15	NaBH(OAc)_3_	Formalin	50	47
1	10	20	30	NaBH_4_	Formalin	Mixture of products	
1	10	20	30	NaBH_3_CN	Formalin	Mixture of products	
1	10	20	30	NaBH(OAc)_3_	Paraformaldehyde	No reaction	

### *N*-methylation of secondary amines

In this study, we synthesized several *N*-methylated amine derivatives employing the procedure developed in the optimization process. Because of the use of formalin, the reaction can be classified as the LAG technique with η ranging from 0.13 to 0.16, depending on the mass of the substrate. Using an infrared pyrometer, we determined that during the milling process, the temperature increased by approximately 3 °C relative to room temperature. The products were extracted with a minimal amount of dichloromethane, and isolation was performed by flash chromatography. Most of the products consisted of *N*-benzylaniline motifs and were differentiated by the presence of various functional groups. The results are summarized in Table 2.

**Table 2:** *N*-methylation of secondary amines.


Entry	R^1^	R^2^	**3**	Yield (%)^a^
1	C_6_H_5_-CH_2_-	C_6_H_5_-	**a**	89
2	4-Cl-C_6_H_4_-	C_6_H_5_-	**c**	93
3	3-OCH_3_-4-OH-C_6_H_3_-	C_6_H_5_-	**d**	31
4	C_6_H_5_-CH_2_-	4-F-C_6_H_4_-	**f**	88
5	4-Cl-C_6_H_4_-CH_2_-	4-F-C_6_H_4_-	**g**	92
6	4-Cl-C_6_H_4_-CH_2_-	4-Br-C_6_H_4_-	**h**	78
7	4-Cl-C_6_H_4_-CH_2_-	4-Cl-C_6_H_4_-	**i**	82
8	4-Cl-C_6_H_4_-CH_2_-	3,5-di-CH_3_-C_6_H_3_-	**k**	95
9	C_6_H_5_-CH_2_-	3,5-di-CH_3_-C_6_H_3_-	**j**	91
10	2-HO-C_6_H_4_-CH_2_-	4-CH_3_-C_6_H_4_-	**m**	76
11		4-F-C_6_H_4_-	**q**	89
12		C_6_H_5_-	**r**	92
13	C_6_H_5_-CH_2_-	C_6_H_5_-CH_2_-	**s**	84
14	4-Cl-C_6_H_4_-CH_2_-	C_6_H_5_-CH_2_-	**t**	88
15	2-HO-C_6_H_4_-CH_2_-	C_6_H_5_-CH_2_-	**u**	68
16	C_6_H_5_-CH_2_-	Cyclohexyl-	**v**	82
17	4-Cl-C_6_H_4_-CH_2_-	Cyclohexyl-	**w**	83
18	2-HO-C_6_H_4_-CH_2_-	Cyclohexyl-	**x**	62
19			**y**	78

**1** (0.5 mmol), Na_2_CO_3_ (0.5 mmol), **2** formalin (1 mmol), NaBH(OAc)_3_ (1.5 mmol), 10 mm ball, milling 30 Hz, 20 min.

^a^Isolated yield.

The mechanochemical procedure was successfully applied for the synthesis of *N*-methylated aromatic-aliphatic and aliphatic-aliphatic tertiary amines. Our research found that the developed new method was characterized by high yields ranging from 78 to 95%, regardless of the functional groups used. An exception was compounds having a hydroxyl group in their structure, for which a decrease in reaction efficiency was observed (Table 2, entries 3, 10, 15, 18). In these cases, the lower reaction yield may be caused by undesired aromatic electrophilic substitution reactions and subsequent reactions typical for strongly activated aromatic rings, resulting in the formation of by-products that were difficult to isolate and identify. For this reason, we were not also able to perform successful *N*-methylation of strongly activated benzene rings such as 3,5-dimethoxyaniline derivatives. Such reactions lead to the formation of complicated, red-colored reaction mixtures that cannot be separated due to the clogging of chromatographic columns. Additionally, the 4-iodoaniline derivatives could not be employed for mechanochemical *N*-methylation reactions because they tended to decompose under ball-milling conditions.

Reaction mixtures, such as those described in our work, usually cause difficulties in the selection of proper solvents in regard to conventional methods. While even slightly nonpolar organic solvents, e.g., acetonitrile, are not suitable for dissolving inorganic compounds, more polar solvents, such as methanol, undergo a reaction with the reducing agent, rendering it deactivated^24^. The only polar solvent available for such reactions is acetic acid, which is troublesome for further processing of the mixture. Other solvents that may be used in a specific case are 1,2-dichloroethane and THF. It should be emphasized that the developed procedure fits perfectly not only for the needs of environmentally friendly synthesis but also solves technical difficulties. Therefore, a new optimized mechanochemical procedure may be a great alternative to conventional solvent-based methods, allowing for the effective preparation of *N*-methylation products in a short time.

### The study on selectivity

During our studies, we encountered unexpected outcomes of our reactions. The issue was found in the ball-milling substances that consisted of a hydroxy group in position 2 of a benzyl ring in *N*-benzylaniline derivatives. The study revealed that such compounds are able to form ring closure products as well as desired *N*-methylation products. Further investigation was carried out to determine if conducting such reactions in a conventional way leads to different selectivity when compared with that in the mechanochemical procedure. The results are summarized in Table 3.

**Table 3:** Study of selectivity between ball-milling and solvent-based synthesis.

				
Entry	Substrate **1**		Mixer Mill		Solution	
			Yield **3** (%)	Yield **4** (%)	Yield **3** (%)	Yield **4** (%)
1		**e**	88	0	48	37
2		**n**	75	13	23	65
3		**p**	48	44	18	74
4		**o**	28	67	19	73
5		**z**	74	0	70	18
6		**b**	84	0	66	20
7		**l**	70	0	58	17

**Figure 1:**
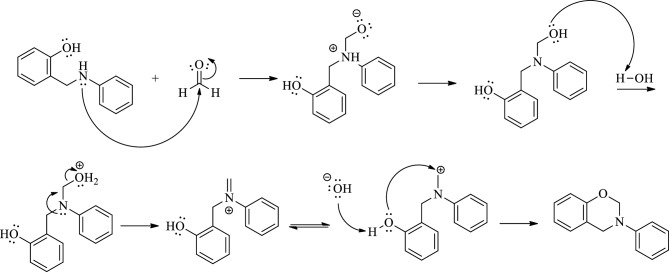
The plausible mechanism of 3-phenyl-3,4-dihydro-2*H*-benzo[e][1,3]oxazine synthesis.

On the basis of the results from this study, it can be concluded that the selectivity of ball milling indeed differs from that of the solvent-based procedure, as illustrated in Table 3. Overall, the conventional synthesis has selectivity shifted toward ring closure products **4** stronger than the mechanochemical approach. The formation of products **4** can be reduced or avoided (Table 3 entries 1, 5–7) under mechanochemical conditions. Such an outcome can be justified because under ball-milling conditions, the reagents have better contact with each other due to a lack of solvation. Consequently, the reducing agent can react with the formed iminium cation. In the case of reaction in solution, the reduction is slower, especially when a weak reducing agent used; thus, the intermediate has a higher chance of reacting with the hydroxyl group. The fact that ring closure products are suggested to form when reacting the corresponding secondary hydroxy-amine with formaldehyde in the absence of a reducing agent, at least partially, supports the formation mechanism of cyclic product **4**^25^. The plausible mechanism underlying the formation of cyclic product **4** is presented in Fig. 1.

## Conclusions

In the present work, we described our research on the development of an efficient, solvent-free, mechanochemical synthetic procedure for the *N-*methylation of secondary amines. The procedure was an example of reductive amination that involved the use of formaldehyde and a reducing agent. This study was performed to determine optimal milling parameters, such as the number and diameter of the milling balls, milling time and frequency, and the form of formaldehyde and borohydride. The optimal conditions were then applied to perform *N*-methylation of various secondary amines with 78–95% yields. An exception was compounds having a hydroxyl group in their structure, for which a decrease in reaction efficiency was observed. In this case, the method's limitations are probably related to the occurrence of undesired aromatic electrophilic substitution reactions and subsequent reactions typical for strongly activated aromatic rings with increased electron density. Furthermore, this study was carried out to investigate the different selectivities of the *N-*methylation reaction in the case of ball-milling and solvent-based synthesis. The mechanochemical synthesis allowed for conducting the reactions in a solid state, which highly facilitates the reaction as a result of the elimination of solvents from the reaction mixtures. The ball-milling technique is also superior to conventional synthesis in regard to selectivity toward* N*-methylated products **3** versus cyclic products **4** (Table 3).

Our results, along with the increasing need for solvent-free environmentally friendly synthetic procedures, may provide a synthetic direction for further development of mechanochemical procedures and for adapting ball-milling conditions for larger scale industrial applications. Since the results of mechanochemical reactions might be totally different from solvent-based synthesis, we highly recommend exploring the possibilities of alternate reaction pathways when applying mechanochemistry for organic synthesis.

### Supplementary Information

Supplementary Information.

## Data Availability

All data generated or analysed during this study are included in this published article and its supplementary information files.
