# Supplementary material for: Convenient and efficient N-methylation of secondary amines under solvent-free ball milling conditions
Source: Sci Rep. 2024 Apr 16;14:8810. doi: 10.1038/s41598-024-59374-z (PMC11021465; doi:10.1038/s41598-024-59374-z)
Supplement: Supplementary file 1 — Supplementary Information. [file 41598_2024_59374_MOESM1_ESM.docx]

**SUPPLEMENTARY INFORMATION**

**Convenient and efficient *N*-methylation of secondary amines under solvent-free ball milling conditions**

**Mikołaj Walter, Olga Ciupak, Karol Biernacki, Janusz Rachoń, Dariusz Witt, Sebastian Demkowicz***

*Department of Organic Chemistry, Faculty of Chemistry, Gdańsk University of Technology, Narutowicza 11/12, 80-233 Gdansk, Poland, e-mail: sebdemko@pg.gda.pl.*

***N*-benzyl-N-methylaniline 3a**
**Chromatography**: PE:DCM (2:1), R_f_ =0,34, yellowish oil, yield 89%,
**^1^H NMR** (500 MHz, DMSO-d_6_) δ 7.38 – 7.28 (m, 2H), 7.25 – 7.18 (m, 3H), 7.18 – 7.10 (m, 2H), 6.74 – 6.68 (m, 2H), 6.61 (tt, *J* = 7.3, 1.0 Hz, 1H), 4.56 (s, 2H), 3.00 (s, 3H).
**^13^C NMR** (126 MHz, DMSO-d_6_) δ 129.45, 129.42, 129.26, 128.86, 128.82, 127.17, 127.10, 127.04, 116.30, 114.41, 112.47, 55.78, 39.00.
**IR (ATR)**: 2916 (w), 1596 (s), 1477 (m), 1245 (m), 837 (s), 625 (s), 456 (s)
**HRMS (ESI):** m/z [M + H]^+^ calcd for C_14_H_16_N: 198.1277; found: 198.1278.

**Figure S1. ^1^H NMR spectrum of 3a**

**Figure S2. ^13^C NMR spectrum of 3a**

**Figure S3. IR spectrum of**

***N*-(2-hydroxybenzyl)-*N*-methylaniline** **3b:**

**Chromatography**: PE:DCM (1:1), R_f_ =0,37, yellowish oil, yield 84%,
**^1^H NMR** (500 MHz, DMSO-d_6_) δ 9.57 (s, 1H), 7.16 – 7.09 (m, 2H), 7.04 (td, *J* = 7.6, 1.7 Hz, 1H), 6.85 (ddd, *J* = 16.5, 7.8, 1.4 Hz, 2H), 6.72 – 6.62 (m, 3H), 6.58 (t, *J* = 7.2 Hz, 1H), 4.45 (s, 2H), 3.02 (s, 3H). **^13^C NMR** (126 MHz, DMSO-d_6_) δ 149.12, 140.77, 129.27, 128.72, 127.62, 127.04, 116.17, 112.72, 46.91, 39.63
**IR (ATR):** 3414 (w), 3026 (w), 2854 (w), 1597 (s), 1504 (m), 1352 (s), 727 (s), 690 (s)
**HRMS (ESI):** m/z [M + H]^+^ calcd for C_14_H_16_NO: 214.1226; found: 214.1225.

**Figure S4. ^1^H NMR spectrum of 3b**

**Figure S5. ^13^C NMR spectrum of 3b**

**Figure S6 IR spectrum of 3b**

***N*-(4-chlorobenzyl)-*N*-methylaniline 3c

Chromatography:** PE:DCM (2:1), R_f_ =0,32, yellowish oil, yield 93%,
**^1^H NMR** (500 MHz, DMSO-d_6_) δ 7.40 – 7.34 (m, 2H), 7.25 – 7.19 (m, 2H), 7.18 – 7.10 (m, 2H), 6.73 – 6.67 (m, 2H), 6.62 (tt, *J* = 7.3, 1.1 Hz, 1H), 4.55 (s, 2H), 3.00 (s, 3H).
**^13^C NMR** (126 MHz, DMSO-d_6_) δ 149.53, 139.56, 129.42, 128.86, 127.17, 127.09, 116.29, 112.46, 55.77, 39.00.
**IR (ATR):** 3026 (w), 2892 (w), 1598 (s), 1488 (m), 1224 (m), 940 (s), 748 (s), 691 (s).
**HRMS (ESI):** m/z [M + H]^+^ calcd for C_14_H_15_ClN: 232.0888; found: 232.0891

**Figure S7. ^1^H NMR spectrum of 3c**

**Figure S 8. ^13^C NMR spectrum of 3c**

**Figure S9. IR spectrum for 3c**

***N-*(4-hydroxy-3-methoxybenzyl)-*N*-methylaniline 3d:

Chromatography:** PE:DCM (1:1), R_f_ =0,37, yellowish oil, yield 31%,
**^1^H NMR** (500 MHz, DMSO-d_6_) δ 8.82 (s, 1H), 7.18 – 7.10 (m, 2H), 6.78 (d, *J* = 2.0 Hz, 1H), 6.76 – 6.70 (m, 2H), 6.69 (d, *J* = 8.0 Hz, 1H), 6.64 – 6.55 (m, 2H), 4.42 (s, 2H), 3.70 (s, 3H), 2.95 (s, 3H).
**^13^C NMR** (126 MHz, DMSO-d_6_) δ 149.78, 148.03, 145.78, 130.08, 129.35, 129.28, 119.58, 116.28, 115.77, 112.72, 111.62, 55.94, 55.72, 38.79.
**IR (ATR):** 3412 (w), 3049 (w), 2842 (w), 1599 (s), 1504 (s), 1429 (s), 1264 (m), 1230(m), 793 (m)
**HRMS (ESI):** m/z [M + H]^+^ calcd for C_15_H_18_NO_2_: 244.1332; found: 244.1335.

**Figure S10. ^1^H NMR spectrum of 3d**

**Figure S11. ^13^C NMR spectrum of 3d**

**Figure S 12. IR spectrum for 3d**

***N*-(5-bromo-2-hydroxybenzyl)-*N*-methylaniline** **3e:

Chromatography:** PE:DCM (1:1), R_f_ =0,32, yellowish oil, yield 88%,
**^1^H NMR** (500 MHz, DMSO-d_6_) δ 9.97 (s, 1H), 7.21 (dd, *J* = 8.6, 2.6 Hz, 1H), 7.19 – 7.11 (m, 2H), 6.95 (d, *J* = 2.5 Hz, 1H), 6.81 (d, *J* = 8.5 Hz, 1H), 6.63 (ddt, *J* = 15.5, 7.3, 1.1 Hz, 3H), 4.43 (s, 2H), 3.02 (s, 3H).
**^13^C NMR** (126 MHz, ) δ 155.54, 149.58, 129.38, 127.86, 127.56, 124.84, 119.13, 115.94, 115.41, 112.08, 51.12, 39.05.  **IR (ATR):** 3422 (w), 3029 (w), 2925 (w), 1506 (s), 1452 (s), 1221 (s), 815 (s), 734 (s), 694 (s)
**HRMS (ESI):** m/z [M + H]^+^ calcd for C_14_H_15_BrNO: 292.0332; found: 292.0329.

**Figure S13. ^1^H NMR spectrum of 3e**

**Figure S14. ^13^C NMR spectrum of 3e**

**Figure S15. IR spectrum of 3e**

***N*-benzyl-*N*-methyl-4-fluoroaniline 3f:

Chromatography:** PE:DCM (2:1), R_f_ =0.3, yellowish oil, yield 88%, **^1^H NMR** (500 MHz, DMSO-d_6_) δ 7.31 (dd, *J* = 8.2, 6.9 Hz, 2H), 7.26 – 7.17 (m, 3H), 7.03 – 6.94 (m, 2H), 6.74 – 6.66 (m, 2H), 4.52 (s, 2H), 2.97 (s, 3H) .
**^13^C NMR** (126 MHz, DMSO-d_6_) δ 149.53, 139.56, 129.45, 129.42, 129.25, 128.86, 128.82, 128.70, 127.61, 127.17, 127.09, 127.03, 127.01, 116.29, 114.39, 112.70, 112.46, 55.77, 39.00.
**IR (ATR):** 2854 (w), 1599 (s), 1478 (s), 1245 (m), 1093 (m), 926 (m), 749 (m), 692 (s)
**HRMS (ESI):** m/z [M + H]^+^ calcd for C_14_H_15_FN: 216.1183; found: 216.1185.

**Figure S16. ^1^H NMR spectrum of 3f**

**Figure S17. ^13^C NMR spectrum of 3b**

**Figure S18. IR spectrum of 3f**

***N*-(4-chlorobenzyl)-*N*-methyl-4-fluoroaniline 3g:

Chromatography:** PE:DCM (2:1), R_f_ =0.32, yellowish oil, yield 92%,
**^1^H NMR** (500 MHz, DMSO-d_6_) δ 7.40 – 7.34 (m, 2H), 7.24 – 7.19 (m, 2H), 7.03 – 6.94 (m, 2H), 6.73 – 6.65 (m, 2H), 4.51 (s, 2H), 2.96 (s, 3H).
**^13^C NMR** (126 MHz, DMSO-d_6_) δ 155.98, 146.37, 138.45, 131.74, 129.20, 128.83, 113.93, 113.87, 55.86, 39.43.
**IR (ATR):** 2879 (w), 1506 (s), 1489 (s), 1224 (s), 1013 (s), 807 (s), 482 (s)
**HRMS (ESI):** m/z [M + H]^+^ calcd for C_14_H_14_ClFN: 250.0793; found: 250.0790.

**Figure S19. ^1^H NMR spectrum of 3g**

**Figure S20. ^13^C NMR spectrum of 3g**

**Figure S21. IR spectrum of 3g**

***N*-(4-chlorobenzyl)-*N*-methyl-4-bromoaniline 3h:

Chromatography:** PE:DCM (2:1), R_f_ =0.32, white waxy solid, yield 78%,
**^1^H NMR** (500 MHz, DMSO-d_6_) δ 7.41 – 7.34 (m, 2H), 7.30 – 7.23 (m, 2H), 7.23 – 7.17 (m, 2H), 6.67 – 6.62 (m, 2H), 4.56 (s, 2H), 3.00 (s, 3H).
**^13^C NMR** (126 MHz, DMSO-d_6_) δ 148.46, 146.52, 138.12, 131.93, 131.87, 129.01, 128.84, 54.74, 39.15. **IR (ATR):** 3029 (w), 2848 (w), 1590 (s), 1487 (m), 1227 (m), 1013 (s), 810 (m), 498 (m)
**HRMS (ESI):** m/z [M + H]^+^ calcd for C_14_H_14_BrClN: 309.9993; found: 309.9989.

**Figure S22. ^1^H NMR spectrum of 3h**

**Figure S23. 13C NMR spectrum of 3h**

**Figure S24. IR spectrum of 3h**

***N*-(4-chlorobenzyl)-*N*-methyl-4-chloroaniline 3i:

Chromatography:** PE:DCM (2:1), R_f_ =0.33, yellowish oil, yield 82%,
**^1^H NMR** (500 MHz, DMSO-d_6_) δ 7.41 – 7.34 (m, 2H), 7.23 – 7.10 (m, 4H), 6.74 – 6.67 (m, 2H), 4.56 (s, 2H), 3.00 (s, 3H).
**^13^C NMR** (126 MHz, DMSO-d_6_) δ 148.46, 146.52, 138.12, 131.93, 131.87, 129.02, 128.90, 114.52, 54.74, 39.15.
**IR (ATR):** 3027 (w), 2785 (m), 1489 (s), 1452 (s), 1363 (s), 1087 (s), 1014 (s), 798 (s), 736 (s), 696 (s)
**HRMS (ESI):** m/z [M + H]^+^ calcd for C_14_H_14_Cl_2_N: 266.0498; found: 266.0497

**Figure S25. 1H NMR spectrum of 3i**

**Figure S26. ^13^C NMR spectrum of 3i**

**Figure S27. IR spectrum of 3i**

***N*-benzyl-*N*-methyl-3,5-dimethylaniline** **3j:

Chromatography:** PE:DCM (2:1), R_f_ =0.36, yellowish oil, yield 91%,
**^1^H NMR** (500 MHz, DMSO-d_6_) δ 7.34 – 7.27 (m, 2H), 7.25 – 7.16 (m, 3H), 6.35 (s, 2H), 6.27 (s, 1H), 4.53 (s, 2H), 2.94 (s, 3H), 2.16 (s, 6H).
**^13^C NMR** (126 MHz, DMSO-d_6_) δ 149.87, 139.73, 138.18, 128.84, 127.23, 127.07, 118.49, 110.58, 55.81, 38.86, 21.94.
**IR (ATR):** 3409 (w), 3027 (w), 2914 (w), 1598 (s), 1451 (s), 1335 (s), 1181 (s), 819 (s), 731 (s), 689 (s)
**HRMS (ESI):** m/z [M + H]^+^ calcd for C_16_H_20_N: 226.1590; found: 226.1593.

**Figure S28. ^1^H NMR spectrum of 3j**

**Figure S29. ^13^C NMR spectrum of 3j**

**Figure S30. IR spectrum of 3j**

***N*-(4-chlorobenzyl)-*N*-methyl-3,5-dimethylaniline** **3k:

Chromatography:** PE:DCM (2:1), R_f_ =0.35, yellowish oil, yield 95%,
**^1^H NMR** (500 MHz, DMSO-d_6_) δ 7.40 – 7.34 (m, 2H), 7.23 – 7.17 (m, 2H), 6.34 (s, 2H), 6.28 (s, 1H), 4.52 (s, 2H), 2.94 (s, 3H), 2.16 (s, 6H).
**^13^C NMR** (126 MHz, DMSO-d_6_) δ 149.59, 138.79, 138.25, 131.59, 129.07, 128.80, 118.64, 110.57, 55.12, 38.87, 21.92.
**IR (ATR):** 2916 (w), 1597 (s), 1488 (s), 1334 (s), 1088 (s), 1013 (s), 817(m), 689 (s), 479 (s)
**HRMS (ESI):** m/z [M + H]^+^ calcd for C_16_H_19_ClN: 260.1201; found: 260.1205.

**Figure S31. ^1^H NMR spectrum of 3k**

**Figure S32. 13C NMR spectrum of 3k**

**Figure S33. IR spectrum of 3k**

***N*-(2-hydroxybenzyl)-*N*-methyl-3,5-dimethylaniline** **3l:

Chromatography:** PE:DCM (1:1), R_f_ =0.31, yellowish oil, yield 70%,
**^1^H NMR** (500 MHz, DMSO-d_6_) δ 9.54 (s, 1H), 7.03 (td, *J* = 7.7, 1.7 Hz, 1H), 6.84 (ddd, *J* = 12.1, 7.8, 1.4 Hz, 2H), 6.69 (td, *J* = 7.4, 1.2 Hz, 1H), 6.33 (d, *J* = 11.1 Hz, 0H), 6.29 (s, 2H), 6.25 (s, 1H), 4.42 (s, 2H), 2.96 (s, 3H), 2.15 (s, 6H).
**^13^C NMR** (126 MHz, DMSO-d_6_) δ 155.50, 149.88, 138.11, 127.82, 127.57, 125.06, 119.19, 118.15, 115.38, 110.14, 51.10, 38.98, 21.97.
**IR (ATR):** 3027 (w), 2785 (m), 1489 (s), 1452 (s), 1363 (s), 1087 (s), 1014 (s), 798 (s), 736 (s), 696 (s)
**HRMS (ESI):** m/z [M + H]^+^ calcd for C_16_H_20_NO: 242.1539; found: 242.1541.

**Figure S34. ^1^H NMR spectrum of 3l**

**Figure S35. ^13^C NMR spectrum of 3l**

**Figure S36. IR spectrum of 3l**

***N*-(2-hydroxybenzyl)-*N*-methyl-4-methylaniline** **3m:

Chromatography:** PE:DCM (1:1), R_f_ =0.32, yellowish oil, yield 76%,
**^1^H NMR** (500 MHz, DMSO-d_6_) δ 9.56 (s, 1H), 7.08 – 6.97 (m, 2H), 6.85 (ddd, *J* = 14.3, 7.8, 1.4 Hz, 2H), 6.69 (td, *J* = 7.4, 1.1 Hz, 1H), 6.49 (t, *J* = 1.9 Hz, 1H), 6.48 – 6.39 (m, 2H), 4.44 (s, 2H), 2.99 (s, 3H), 2.20 (s, 3H).
**^13^C NMR** (126 MHz, DMSO-d_6_) δ 155.50, 149.74, 138.32, 129.25, 127.84, 127.58, 124.97, 119.18, 116.97, 115.39, 112.70, 109.49, 51.11, 39.03, 22.07.
**IR (ATR):** 2920 (w), 1588 (m), 1489 (s), 1445 (s), 1225 (m), 751 (s), 692 (s)
**HRMS (ESI):** m/z [M + H]^+^ calcd for C_15_H_18_NO: 228.1383; found: 228.1381.

**Figure S37. ^1^H NMR spectrum of 3m**

**Figure S38. ^13^C NMR spectrum of 3m**

**Figure S39. IR spectrum of 3m**

***N*-(5-bromo-2-hydroxybenzyl)-*N*-methyl-4-fluoroaniline** **3n:

Chromatography:** PE:DCM (1:1), R_f_ =0.3, yellowish oil, yield 75%,
**^1^H NMR** (500 MHz, DMSO-d_6_) δ 9.97 (s, 1H), 7.22 (dd, *J* = 8.5, 2.6 Hz, 1H), 7.07 – 6.96 (m, 3H), 6.81 (d, *J* = 8.5 Hz, 1H), 6.75 – 6.68 (m, 0H), 6.67 – 6.59 (m, 2H), 4.40 (s, 2H), 2.99 (s, 3H).
**^13^C NMR** (126 MHz, DMSO-d_6_) δ 155.82, 153.97, 146.49, 139.36, 128.87, 128.66, 127.27, 127.16, 126.94, 115.80, 115.63, 113.72, 113.66, 56.41, 39.44.
**IR (ATR):** 3244 (w), 2924 (w), 1506 (s), 1477 (m), 1420 (s), 1243 (m), 1106 (m), 815 (m), 623 (s), 532 (s)
**HRMS (ESI):** m/z [M + H]^+^ calcd for C_14_H_14_BrFNO: 310.0237; found 310.0238.

**Figure S40. ^1^H NMR spectrum of 3n**

**Figure S41. ^13^C NMR spectrum of 3n**

**Figure S42. IR spectrum of 3n**

***N*-(5-bromo-2-hydroxybenzyl)-*N*-methyl-4-bromoaniline 3o:

Chromatography:** PE:DCM (1:1), R_f_ =0.32, yellowish oil, yield 23%,
**^1^H NMR** (500 MHz, DMSO-d_6_) δ 10.01 (s, 1H), 7.31 – 7.25 (m, 2H), 7.22 (dd, *J* = 8.6, 2.6 Hz, 1H), 6.92 (d, *J* = 2.5 Hz, 1H), 6.81 (d, *J* = 8.5 Hz, 1H), 6.62 – 6.56 (m, 2H), 4.43 (s, 2H), 3.02 (s, 3H).
**^13^C NMR** (126 MHz, DMSO-d_6_) δ 154.87, 148.50, 131.96, 130.68, 129.63, 127.43, 117.64, 114.15, 110.53, 107.40, 51.02, 39.20.
**IR ATR:** 2960 (w), 1579 (s), 1474 (m), 1248 (m), 1048 (m), 1005 (s), 823 (s), 669 (s), 532(s) **HRMS (ESI):** m/z [M + H]^+^ calcd for C_14_H_14_Br_2_NO: 369.9437; found 369.9441.

**Figure S43. 1H NMR spectrum of 3o**

**Figure S44. ^13^C NMR spectrum of 3o**

**Figure S45. IR spectrum of 3o**

***N*-(5-bromo-2-hydroxybenzyl)-*N*-methyl-4-chloroaniline** **3p:

Chromatography:** PE:DCM (1:1), R_f_ =0.32, yellowish oil, yield 48%,
**^1^H NMR** (500 MHz, DMSO-d_6_) δ 10.01 (s, 1H), 7.22 (dd, *J* = 8.5, 2.6 Hz, 1H), 7.20 – 7.14 (m, 1H), 7.18 – 7.10 (m, 1H), 7.01 – 6.91 (m, 1H), 6.81 (d, *J* = 8.5 Hz, 1H), 6.67 – 6.60 (m, 2H), 4.43 (s, 2H), 3.02 (s, 3H).
**^13^C NMR** (126 MHz, DMSO-d_6_) δ 155.08, 148.18, 130.63, 129.61, 129.13, 127.48, 119.93, 117.68, 113.57, 110.36, 51.13, 39.24.
**IR ATR:** 2923 (w), 1593 (m), 1480 (m), 1221 (m), 935 (s), 807 (m), 607 (s), 526 (s)
**HRMS (ESI):** m/z [M + H]^+^ calcd for C_14_H_14_BrClNO: 325.9942; found 325.9944.

**Figure S46. ^1^H NMR spectrum of 3p**

**Figure S47. ^13^C NMR spectrum of 3p**

**Figure S48. IR spectrum of 3p**

***N*-methyl-*N*-(3-pirydynylmethyl)-4-fluoroaniline** **3q:

Chromatography:** DCM, R_f_ =0.32, yellowish oil, yield 89%,
**^1^H NMR** (500 MHz, DMSO-d_6_) δ 8.47 – 8.42 (m, 2H), 7.59 (ddd, *J* = 7.8, 2.4, 1.6 Hz, 1H), 7.33 (ddd, *J* = 7.8, 4.8, 0.9 Hz, 1H), 7.05 – 6.96 (m, 2H), 6.78 – 6.70 (m, 2H), 4.57 (s, 2H), 2.96 (s, 3H).
**^13^C NMR** (126 MHz, DMSO-d_6_) δ 156.10, 149.09, 148.57, 146.32, 146.31, 135.28, 123.96, 115.89, 115.72, 54.13, 39.38.
**IR (ATR):** 3055 (w), 2877 (w), 1506 (s), 1425 (s), 1200 (s), 817 (m), 709 (s), 510 (s)
**HRMS (ESI):** m/z [M + H]^+^ calcd for C_13_H_14_FN_2_: 217.1136; found 217.1135.

**Figure S49. ^1^H NMR spectrum of 3q**

**Figure S50. 13C NMR spectrum of 3q**

**Figure S51. IR spectrum of 3q**

***N*-methyl-*N*-(2-pirydynylmethyl)aniline** **3r:

Chromatography:** DCM, R_f_ =0.35, yellowish oil, yield 92%,
**^1^H NMR** (500 MHz, DMSO-d_6_) δ 8.54 (ddd, *J* = 4.9, 1.8, 0.9 Hz, 1H), 7.70 (td, *J* = 7.7, 1.8 Hz, 1H), 7.27 – 7.21 (m, 1H), 7.17 – 7.09 (m, 3H), 6.72 6.65 (m, 2H), 6.60 (tt, *J* = 7.3, 1.1 Hz, 1H), 4.63 (s, 2H), 3.09 (s, 3H).
**^13^C NMR** (126 MHz, DMSO-d_6_) δ 160.27, 149.34, 148.87, 137.06, 129.34, 122.41, 121.51, 116.39, 112.71, 48.97, 39.77.
**IR (ATR):** 3026 (w), 2878 (w), 1597 (m), 1488 (m), 1012 (s), 805 (s), 747 (s), 690 (s), 480 (s)
**HRMS (ESI):** m/z [M + H]^+^ calcd for C_13_H_15_N_2_: 199.1230; found 199.1232.

**Figure S52. ^1^H NMR spectrum of 3r**

**Figure S53. ^13^C NMR spectrum of 3r**

**Figure S54. IR spectrum of 3r**

***N*-benzyl-*N*-methylbenzylamine 3s:

Chromatography:** PE:DCM (1:1), R_f_ =0.34, yellowish oil, yield 84%,
**^1^H NMR** (500 MHz, DMSO-d_6_) δ 7.37 – 7.31 (m, 7H), 7.28 – 7.23 (m, 2H), 3.49 (s, 4H), 2.07 (s, 3H).
**^13^C NMR** (126 MHz, DMSO-d_6_) δ 139.54, 129.01, 128.64, 127.32, 61.46, 42.12.
**IR (ATR):** 3026 (w), 2783 (w), 1494 (s), 1451 (s), 1365 (s), 1023 (s), 732 (s), 695 (s)
**HRMS (ESI):** m/z [M + H]^+^ calcd for C_15_H_18_N: 212.1434; found 212.1436.

**Figure S55. ^1^H NMR spectrum of 3s**

**Figure S56. ^13^C NMR spectrum of 3s**

**Figure S57. IR spectrum of 3s**

***N*-(4-chlorobenzyl)-*N*-methylbenzylamine 3t:

Chromatography:** PE:DCM (1:1), R_f_ =0.36, yellowish oil, yield 88%,
**^1^H NMR** (500 MHz, DMSO-d_6_) δ 7.48 – 7.19 (m, 9H), 3.49 (s, 2H), 3.48 (s, 2H), 2.06 (s, 3H).
**^13^C NMR** (126 MHz, DMSO-d_6_) δ 139.38, 138.61, 130.75, 129.02, 128.65, 128.61, 127.37, 61.43, 60.54, 42.02.
**IR (ATR):** 3028 (w), 2786 (m), 1490 (s), 1452 (s), 1364 (s), 1025 (s), 798 (s),697 (m), 451 (s)
**HRMS (ESI):** m/z [M + H]^+^ calcd for C_15_H_17_ClN: 246.1044; found 246.1046.

**Figure S58. 1H NMR spectrum of 3t**

**Figure S59. ^13^C NMR spectrum of 3t**

**Figure S60. IR spectrum of 3t**

***N*-(2-hydroxybenzyl)-*N*-methylbenzylamine** **3u:

Chromatography:** DCM, R_f_ =0.32, yellowish oil, yield 68%,
**^1^H NMR** (500 MHz, DMSO-d_6_) δ 10.59 (s, 1H), 7.41 – 7.25 (m, 5H), 7.14 (dd, *J* = 7.5, 1.7 Hz, 1H), 7.10 (td, *J* = 7.7, 1.7 Hz, 1H), 6.80 – 6.72 (m, 2H), 3.66 (s, 2H), 3.57 (s, 2H), 2.13 (s, 3H).
**^13^C NMR** (126 MHz, DMSO-d_6_) δ 166.61, 157.27, 138.25, 129.49, 129.42, 128.83, 128.65, 128.59, 127.72, 123.35, 119.26, 115.74, 61.02, 58.45, 41.42.
**IR (ATR):** 3028 (w), 2794 (w), 1588 (s), 1475 (m), 1453 (m), 1253 (s), 1009 (s), 743 (s), 697 (s)
**HRMS (ESI):** m/z [M + H]^+^ calcd for C_15_H_18_NO: 228.1383; found 228.1385.

**Figure S61. ^1^H NMR spectrum of 3u**

**Figure S62. ^13^C NMR spectrum of 3u**

**Figure S63. IR spectrum of 3u**

***N*-benzyl-*N*-methyl-cyclohexylamine** **3v:

Chromatography:** PE:DCM (2:1), R_f_ =0.34, yellowish oil, yield 82%,
**^1^H NMR** (500 MHz, DMSO-d_6_) δ 7.30 (d, *J* = 5.4 Hz, 4H), 7.25 – 7.19 (m, 1H), 3.53 (s, 2H), 2.39 (tt, *J* = 11.4, 3.2 Hz, 1H), 2.09 (s, 3H), 1.83 – 1.70 (m, 4H), 1.63 – 1.55 (m, 1H), 1.33 – 1.13 (m, 4H), 1.08 (qt, *J* = 12.4, 3.0 Hz, 1H).
**^13^C NMR** (126 MHz, DMSO-d_6_) δ 131.89, 128.80, 128.50, 127.04, 97.26, 62.20, 57.75, 39.79, 37.50, 28.55, 26.38, 25.93.
**IR (ATR):** 2926 (s), 2852 (s), 2783 (s), 1494 (s), 1450 (s), 1026 (m), 733 (s), 696 (s)
**HRMS (ESI):** m/z [M + H]^+^ calcd for C_14_H_22_N: 204.1747; found 204.1748.

**Figure S64. ^1^H NMR spectrum of 3v**

**Figure S65. ^13^C NMR spectrum of 3v**

******

**Figure S66. IR spectrum of 3v**

***N*-(4-chlorobenzyl)-*N*-methyl-cyclohexylamine** **3w:

Chromatography:** PE:DCM (2:1), R_f_ =0.35, yellowish oil, yield 83%,
**^1^H NMR** (500 MHz, DMSO-d_6_) δ 7.38 – 7.30 (m, 4H), 3.52 (s, 2H), 2.38 (tt, *J* = 11.4, 3.2 Hz, 1H), 2.08 (s, 3H), 1.82 – 1.70 (m, 4H), 1.62 – 1.53 (m, 1H), 1.33 – 1.12 (m, 4H), 1.08 (ddt, *J* = 15.5, 12.3, 6.1 Hz, 1H).
**^13^C NMR** (126 MHz, DMSO-d_6_) δ 139.83, 131.49, 130.58, 128.47, 62.20, 56.93, 37.54, 28.58, 26.35, 25.91.
**IR (ATR):** 2926 (s), 2852 (s), 2784 (s), 1489 (s), 1449 (s), 1088 (s), 1014 (s), 802 (s), 488 (s)
**HRMS (ESI):** m/z [M + H]^+^ calcd for C_14_H_21_ClN: 238.1357; found 238.1356.

**Figure S67. ^1^H NMR spectrum of 3w**

**Figure S68. 13C NMR spectrum of 3w**

**Figure S69. IR spectrum of 3w**

***N*-(2-hydroxybenzyl)-*N*-methyl-cyclohexylamine 3x:

Chromatography:** PE:DCM (2:1), R_f_ =0.32, yellowish oil, yield 62%,
**^1^H NMR** (500 MHz, DMSO-d_6_) δ 7.11 – 7.06 (m, 1H), 7.04 (dd, *J* = 7.4, 1.6 Hz, 1H), 6.72 (td, *J* = 7.4, 1.2 Hz, 1H), 6.67 (dd, *J* = 8.0, 1.2 Hz, 1H), 3.77 (s, 2H), 2.53 (t, *J* = 3.3 Hz, 1H), 2.19 (s, 3H), 1.83 – 1.73 (m, 4H), 1.63 – 1.56 (m, 1H), 1.32 (qd, *J* = 12.3, 3.6 Hz, 2H), 1.26 – 1.14 (m, 2H), 1.09 (qt, *J* = 12.5, 3.2 Hz, 1H).
**^13^C NMR** (126 MHz, DMSO-d_6_) δ 158.22, 131.90, 131.75, 128.99, 128.46, 123.04, 118.97, 115.71, 61.60, 56.28, 36.58, 27.92, 26.07, 25.72.
**IR (ATR):** 2928 (s), 2854 (w), 1589 (m), 1474 (m), 1450 (m), 1257 (s), 1024 (s), 750 (s), 654 (s)
**HRMS (ESI):** m/z [M + H]^+^ calcd for C_14_H_22_NO: 220.1696; found 220.1698.

**Figure S70. ^1^H NMR spectrum of 3x**

**Figure S71. ^13^C NMR spectrum of 3x**

**Figure S72. IR spectrum of 3x**

**1-(benzo[b]thiophen-4-yl)-4-methylpiperazine 3y:

Chromatography:** PE:DCM (1:1), R_f_ =0.31, white solid, m.p. 101-103°C, yield 78%,
**^1^H NMR** (500 MHz, DMSO-d_6_) δ 7.70 (d, *J* = 5.5 Hz, 1H), 7.64 – 7.59 (m, 1H), 7.40 (dd, *J* = 5.5, 0.9 Hz, 1H), 7.28 (t, *J* = 7.8 Hz, 1H), 6.90 (dd, *J* = 7.7, 0.9 Hz, 1H), 3.07 (d, *J* = 4.7 Hz, 4H), 2.56 (s, 4H), 2.27 (s, 3H).
**^13^C NMR** (126 MHz, DMSO-d_6_) δ 148.70, 140.90, 133.86, 126.28, 125.56, 122.36, 117.11, 112.54, 55.38, 52.02, 46.24.
**IR (ATR):** 2933 (m), 2818 (m), 1561 (s), 1446 (m), 1368 (s), 1286 (s), 1237 (s), 1138 (s), 1009 (s), 965 (m), 749 (m), 558 (m)
**HRMS (ESI):** m/z [M + H]^+^ calcd for C_13_H_17_N_2_S: 233.1107; found 233.1109.

**Figure S73. ^1^H NMR spectrum of 3y**

**Figure S74. ^13^C NMR spectrum of 3y**

**Figure S75. IR spectrum of 3y**

***N*-(5-bromo-2-hydroxybenzyl)-*N*-methyl-3,5-dimethylaniline 3z:

Chromatography:** PE:DCM (1:1), R_f_ =0.34, yellowish oil, yield 74%,
**^1^H NMR** (500 MHz, DMSO-d_6_) δ 10.01 (s, 1H), 7.31 – 7.25 (m, 2H), 7.22 (dd, *J* = 8.5, 2.6 Hz, 1H), 6.92 (d, *J* = 2.5 Hz, 1H), 6.81 (d, *J* = 8.5 Hz, 1H), 6.61 – 6.56 (m, 2H), 4.43 (s, 2H), 3.02 (s, 3H).
**^13^C NMR** (126 MHz, DMSO-d_6_) δ 155.50, 149.88, 138.03, 127.80, 127.57, 125.06, 119.16, 118.15, 115.38, 110.14, 51.10, 38.98, 21.97.
**IR (ATR):** 3345 (w), 2920 (w), 1607 (m), 1589 (m), 1488 (m), 1454 (m), 1245 (m), 1087 (m), 953 (m), 750 (s), 443 (m)
**HRMS (ESI):** m/z [M + H]^+^ calcd for C_16_H_19_BrNO: 320.0645; found 320.0644.

**Figure S76. ^1^H NMR spectrum of 3z**

**Figure S77. ^13^C NMR spectrum of 3z**

**Figure S78. IR spectrum of 3z**

**3-phenyl-3,4-dihydro-2H-benzo[e][1,3]oxazine 4b:

Chromatography:** PE:DCM (1:1), R_f_ =0.38, yellowish solid, m.p. 55-57°C, yield 20%
**^1^H NMR** (500 MHz, DMSO-d_6_) δ 7.25 – 7.19 (m, 2H), 7.15 – 7.10 (m, 2H), 7.10 – 7.02 (m, 1H), 6.84 (tq, *J* = 7.3, 1.2 Hz, 2H), 6.70 (dd, *J* = 8.2, 1.2 Hz, 1H), 5.43 (s, 2H), 4.63 (s, 2H).
**^13^C NMR** (126 MHz, DMSO-d_6_) δ 154.39, 148.24, 129.55, 128.09, 127.63, 121.76, 120.91, 120.87, 117.78, 116.65, 79.08, 49.32.
**IR (ATR):** 3009 (w), 2908 (w), 1600 (s), 1580 (s), 1489 (s), 1454 (m), 1364 (s), 1223 (m), 1152 (s), 932 (m), 752 (m), 690 (s)
**HRMS (ESI):** m/z [M + H]^+^ calcd for C_14_H_14_NO: 212.1070; found: 212.1073.

**Figure S79. ^1^H NMR spectrum of 4b**

**Figure S80. ^13^C NMR spectrum of 4b**

**Figure S81. IR spectrum of 4b**

**6-bromo-3-phenyl-3,4-dihydro-2H-benzo[e][1,3]oxazine 4e:

Chromatography:** PE:DCM (1:1), R_f_ =0.36, yellowish solid, m.p. 57-58°C, yield 37%,
**^1^H NMR** (500 MHz, DMSO-d_6_) δ 7.21 (dd, *J* = 8.6, 2.6 Hz, 1H), 7.18 – 7.12 (m, 2H), 6.95 (d, *J* = 2.5 Hz, 1H), 6.81 (d, *J* = 8.5 Hz, 1H), 6.63 (ddt, *J* = 15.5, 7.3, 1.1 Hz, 3H), 5.46 (s, 2H), 4.67 (s, 2H).
**^13^C NMR** (126 MHz, DMSO-d_6_) δ 153.73, 147.95, 130.81, 130.15, 129.62, 124.29, 121.24, 118.88, 117.98, 112.01, 79.52, 48.86.
**IR (ATR):** 3036 (w), 2910 (w), 1597 (m), 1477 (m), 1363 (s), 1226 (m), 1186 (m), 930 (m), 817 (s), 753 (m), 689 (s), 607 (s)
**HRMS (ESI):** m/z [M + H]^+^ calcd for C_14_H_13_BrNO: 290.0175; found: 290.0173.

**Figure S82. ^1^H NMR spectrum of 4e**

**Figure S83. ^13^C NMR spectrum of 4e**

**Figure S84. IR spectrum of 4e**

**3-(3,5-dimethylphenyl)-3,4-dihydro-2H-benzo[e][1,3]oxazine 4l:

Chromatography:** PE:DCM (1:1), R_f_ =0.38, yellowish solid, m.p. 79-81°C, yield 17%,
**^1^H NMR** (500 MHz, DMSO-d_6_) δ 7.34 – 7.27 (m, 2H), 7.25 – 7.16 (m, 3H), 6.35 (s, 2H), 6.27 (s, 1H), 5.43 (s, 2H), 4.53 (s, 2H), 2.16 (s, 6H).
**^13^C NMR** (126 MHz, DMSO-d_6_) δ 154.47, 148.22, 138.52, 138.26, 128.06, 127.61, 122.58, 122.07, 121.93, 120.83, 116.71, 115.43, 115.19, 78.92, 49.41, 21.69.
**IR (ATR):** 3039 (w), 2911 (w), 1600 (m), 1485 (m), 1454 (m), 1364 (s), 1337 (s), 1223 (s), 1142 (s), 924 (m), 803 (s), 750 (s), 688(s)
**HRMS (ESI):** m/z [M + H]^+^ calcd for C_16_H_18_NO: 240.1383; found: 240.1388.

**Figure S85. ^1^H NMR spectrum of 4l**

**Figure S86. ^13^C NMR spectrum of 4l**

**Figure S87. IR spectrum of 4l**

**6-bromo-3-(4-fluorophenyl)-3,4-dihydro-2H-benzo[e][1,3]oxazine 4n:

Chromatography:** PE:DCM (1:1), R_f_ =0.37, yellowish solid, m.p. 68-70°C, yield 65%,
**^1^H NMR** (500 MHz, DMSO-d_6_) δ 7.22 (dd, *J* = 8.5, 2.6 Hz, 1H), 7.02 – 6.96 (m, 3H), 6.81 (d, *J* = 8.5 Hz, 1H), 6.66 – 6.61 (m, 2H), 5.45 (s, 2H), 4.65 (s, 2H).
**^13^C NMR** (126 MHz, DMSO-d_6_) δ 158.41, 156.51, 153.64, 144.61, 144.59, 130.85, 130.16, 124.03, 120.00, 119.93, 118.86, 112.06, 80.08, 49.34.
**IR (ATR):** 3031 (w), 2849 (w), 1507 (s), 1479 (s), 1453 (m), 1222 (m), 1024 (s), 933 (s), 816 (m), 607 (m)
**HRMS (ESI):** m/z [M + H]^+^ calcd for C_14_H_12_BrFNO: 308.0081; found 308.0078.

**Figure S88. ^1^H NMR spectrum of 4n**

**Figure S89. ^13^C NMR spectrum of 4n**

**Figure S90. IR spectrum of 4n**

**6-bromo-3-(4-bromophenyl)-3,4-dihydro-2H-benzo[e][1,3]oxazine 4o:

Chromatography:** PE:DCM (1:1), R_f_ =0.37, yellowish solid, 70-72°C, yield 73%,
**^1^H NMR** (500 MHz, DMSO-d_6_) δ 7.42 – 7.38 (m, 2H), 7.35 (d, *J* = 2.5 Hz, 1H), 7.25 (dd, *J* = 8.7, 2.5 Hz, 1H), 7.12 – 7.07 (m, 2H), 6.71 (d, *J* = 8.7 Hz, 1H), 5.46 (s, 2H), 4.67 (s, 2H).
**^13^C NMR** (126 MHz, DMSO-d_6_) δ 153.54, 147.23, 132.23, 131.88, 130.89, 130.18, 124.01, 119.99, 118.90, 114.46, 112.72, 112.10, 79.16, 48.74.
**IR (ATR):** 3066 (w), 1587 (s), 1479 (m), 1366 (s), 1221 (s), 1187 (s), 934 (s), 807 (m), 611 (s)
**HRMS (ESI):** m/z [M + H]^+^ calcd for C_14_H_12_Br_2_NO: 367.9280; found 367.9282.

**Figure S91. ^1^H NMR spectrum of 4o**

**Figure S92. ^13^C NMR spectrum of 4o**

**Figure S93. IR spectrum of 4o**

**6-bromo-3-(4-chlorophenyl)-3,4-dihydro-2H-benzo[e][1,3]oxazine 4p:

Chromatography:** PE:DCM (1:1), R_f_ =0.38, yellowish solid, m.p. 94-97°C, yield 74%,
**^1^H NMR** (500 MHz, DMSO-d_6_) δ 7.35 (d, *J* = 2.5 Hz, 1H), 7.31 – 7.23 (m, 3H), 7.17 – 7.12 (m, 2H), 6.71 (d, *J* = 8.7 Hz, 1H), 5.46 (s, 2H), 4.67 (s, 2H).
**^13^C NMR** (126 MHz, DMSO-d_6_) δ 153.54, 147.23, 132.23, 130.89, 130.18, 124.00, 119.98, 118.90, 112.73, 112.11, 79.17, 48.75.
**IR (ATR):** 3068 (w), 1593 (m), 1480 (m), 1364 (s), 1221 (m), 1187 (s), 934 (s), 807 (m), 607 (s)
**HRMS (ESI):** m/z [M + H]^+^ calcd for C_14_H_12_BrClNO: 323.9785; found 323.9788.

**Figure S94. ^1^H NMR spectrum of 4p**

**Figure S95. ^13^C NMR spectrum of 4p**

**Figure S96. IR spectrum of 4p**

**6-bromo-3-(3,5-dimethylphenyl)-3,4-dihydro-2H-benzo[e][1,3]oxazine 4z:

Chromatography:** PE:DCM (1:1), R_f_ =0.39, yellowish oil, yield 18%,
**^1^H NMR** (500 MHz, DMSO-d_6_) δ 7.33 (d, *J* = 2.5 Hz, 1H), 7.24 (dd, *J* = 8.7, 2.5 Hz, 1H), 6.74 (d, *J* = 1.6 Hz, 2H), 6.70 (d, *J* = 8.7 Hz, 1H), 6.51 (s, 1H), 5.43 (s, 2H), 4.64 (s, 2H), 2.20 (s, 6H).
**^13^C NMR** (126 MHz, DMSO-d_6_) δ 153.78, 147.87, 138.59, 130.75, 130.09, 124.44, 122.85, 118.92, 115.54, 111.95, 79.24, 48.91, 21.69.
**IR (ATR):** 2915 (w), 1597 (s), 1477 (s), 1226(s), 934 (s), 811 (m), 735 (s), 691 (s)
**HRMS (ESI):** m/z [M + H]^+^ calcd for C_16_H_17_BrNO: 318.0488; found 318.0486.

**Figure S97. ^1^H NMR spectrum of 4z**

**Figure S98. ^13^C NMR spectrum of 4z**

**Figure S99. IR spectrum of 4z**
